# The availability of six tracer medicines in private medicine outlets in Uganda

**DOI:** 10.1186/s40545-014-0018-y

**Published:** 2014-12-08

**Authors:** Catherine Birabwa, Jude Murison, Valerie Evans, Celestino Obua, Amon Agaba, Paul Waako, Allyson Pollock

**Affiliations:** Department of Pharmacology, Faculty of Medicine, Mbarara University of Technology, Mbarara, Uganda; School of Social and Political Science, University of Edinburgh, Edinburgh, UK; Institute of Development Policy and Management, University of Antwerp, Antwerp, Belgium; Department of Family Medicine, College of Health Sciences, Makerere University, Kampala, Uganda; Department of Pharmacology and Therapeutics, College of Health Sciences, Makerere University, Kampala, Uganda; Centre for Primary Care and Public Health, Queen Mary University of London, London, UK

**Keywords:** Essential medicines, Uganda, Medicine outlet, Pharmacy, Drug shop, Private sector

## Abstract

**Objectives:**

Many low income countries struggle to provide safe and effective medicines due to poor public health care infrastructure, budgetary constraints, and lack of human resource capacity. Private sector pharmacies and drug shops are used by a majority of the population as an alternative to public pharmacies. This study looks at the availability of six essential medicines in private drug outlets across Uganda.

**Methods:**

A standardised medicines availability survey developed by the World Health Organization and Health Action International was adapted for use in this project to collect availability data for six tracer medicines in 126 private medicine outlets across four districts in Uganda from September 2011 to October 2012.

**Results:**

Artemisinin-based combination treatments and metformin were the most commonly found medicines in the private medicine outlets surveyed. Ninty-nine percent of all outlets carried artemisinin-based combinations while 93% of pharmacies and 53% of drug shops stocked metformin. Oxytocin was found in one third of outlets surveyed. Fluoxetine was in 70% of pharmacies yet was not found in any drug shops. Rifampicin and lamivudine were found infrequently in outlets across all districts; 10% and 2%, respectively. Not all brands found in surveyed outlets were listed on the Ugandan National Drug Register. In particular, five unlisted brands of rifampicin were found in private medicine outlets.

**Conclusions:**

The regulatory process should be improved through the enforcement of outlet licensing and medicine registration. Additional studies to elucidate the reasons behind the use of private medicine outlets over the public sector would assist the government in implementing interventions to increase use of public sector medicine outlets.

## Introduction

Medicines availability is a challenge for low income countries due to lack of resources for medicines and health supplies, poor infrastructure and lack of workforce capacity. Following the establishment of the WHO Model Essential Medicines List (EML) in 1977, many countries have adopted this concept in order to prioritise their medicine needs [1]. Under the Ugandan National Drug Policy essential medicines are a means of ensuring that safe, efficacious and good quality medicines are available and accessible at all times and that they are affordable and used appropriately [2]. The Ugandan Ministry of Health’s first national EML list (EMLU) was released in 1991 and is updated approximately every five years, most recently in 2012 [3]. Although essential medicines should be freely available at public health facilities this is not necessarily the case forcing the population to rely on pharmacies and drug shops in the private sector, particularly for obtaining life-saving medicines [4-7]. Few studies have looked at the availability of essential medicines in private outlets. In this study, we conducted a survey of the availability of six tracer medicines listed on the EMLU at private drug retail outlets in Uganda.

### Uganda’s pharmacy and drug retail outlets

The public healthcare system is organised in four tiers: community health teams at the local level, health centres at the parish level, sub-county and constituency up to the district level, and regional and national referral hospitals which can provide comprehensive, specialist care. In the public sector, vertical disease programmes provide medicine consumption data and forecasts for malaria, tuberculosis and HIV/AIDS. However, the private sector makes up nearly 50% of healthcare delivery in Uganda and is subdivided into formal and informal sectors [6,8-10]. The formal sector is comprised of private pharmacies, drug shops, private clinics and private hospitals [2]. The informal sector consists of general merchandise shops and traditional practitioners [11]. Private retailers, both formal and informal, are the major sources of medicines for patients and require licenses from the National Drug Authority (NDA) head office or Regional Inspectors of Drugs [12]).

### Medicine registration and scheduling

Most drugs that are authorised for use in Uganda are listed on the National Drug Register (NDR) [12,13]. Furthermore, the National Drug Policy and Authority Act 1993 provides information about which drugs can be sold by pharmacies and drug shops [14]. Medicines are classified into four schedules: Class A drugs (or narcotics) may be sold by retail only with a prescription and may be supplied only by a registered pharmacist or licensed pharmacy under specific guidelines issued by the NDA; Class B drugs (or controlled drugs) are divided into two groups: group I (prescription-only medicines) may be supplied only with a prescription and group II (pharmacy-initiated medicines) may be supplied without a prescription only by a registered pharmacist or licensed pharmacy; Class C drugs (or licensed/over-the-counter drugs) may be sold by a person or company operating a licensed pharmacy or by a licensed drug seller (according to the specifications of the license); the fourth schedule identifies medicines and articles exempt from regulation by the NDA. While pharmacies can avail all classes of medicines, drug shops are restricted to Class C drugs.

### Pharmacy and drug shop licensing and the law

By law, the NDA must register all drugs shops and pharmacies annually. However, the licensing of pharmacies and drug shops is managed at the regional authority offices. Pharmacies predominantly exist in urban areas, while drug shops are distributed evenly in rural areas [15]. Qualitative studies in Uganda have highlighted problems associated with drug outlets including; inadequate information given to patients, irrational drug sales, or the inappropriate use of medicines and illegal sale of prescription drugs [15-19].

## Methods

The World Health Organization/Health Action International standard survey methodology was used to develop a data collection tool for private sector medicine outlets for six tracer medicines [20]. The six tracer medicines in this study are: artemether-lumefantrine for malaria, metformin for diabetes, oxytocin, a uterotonic used during labour, fluoxetine for mental illness, rifampicin for tuberculosis, and lamivudine for HIV/AIDS. These medicines were chosen to reflect the current disease burden in Uganda for both communicable and non-communicable diseases, and for vertical and non-vertical programmes. All six of these tracer medicines are scheduled as Class B drugs in Uganda.

### Sampling of medicine outlets

Four districts were chosen on the basis of health service delivery performance rankings of districts across Uganda: Kampala (highest performing), Mbarara (mid-upper performing), Apac (mid-low performing) and Bundibugyo (least performing) were selected [21,22]. A list of registered pharmacies in Kampala was obtained from the NDA and a list of drug outlets for Mbarara, Apac and Bundibugyo were obtained from the District Offices. If the selected pharmacy/drug shop was closed or relocated the data collector went to the nearest private medicine outlet. The number of outlets surveyed by district and outlet type is shown in Table 1. Of the 126 outlets, 47 were in Kampala, 37 in Mbarara, 31 in Bundibugyo, and 11 in Apac. Overall, 60 registered pharmacies and 66 drug shops were sampled for this study (Table 1).

**Table 1:** **Country and district demographics; total and surveyed private medicine outlets**

**Region**	**Population coverage** ^**†**^	**Medicine outlet type (outlets surveyed)**	
		Pharmacy*	***Drug Shop***
**Country**	34.0 million	510	5263**
Kampala	1,659,600	103 (45)	Unknown (2)
Mbarara	83,700	15 (9)	Unknown (28)
Bundibugyo	21,600	2 (2)	Unknown (29)
Apac	13,700	0 (4)	Unknown (7)

^†^Mid-year projection 2011 [26]; *NDA Registered Pharmacies [13]; **[9]; surveyed outlets are in parentheses.

### Data collection and analysis

Data were collected from September 2011 to October 2012. One hundred and twenty-six medicine outlets were surveyed and semi-structured questionnaires were administered to the pharmacist or drug shop owner. Survey results were double entered into EpiInfo (v3.5.3) and cleaned by the data manager. Data analysis was carried out with EpiInfo and Microsoft Excel. Descriptive analysis was performed to compare availability of medicines in pharmacies versus drug shops. Availability was calculated using the number of tracer medicines found as a percentage of the total number of surveyed outlets (Table 2 and Table 3). The NDR from 2011 was reviewed to obtain information on total number and individual brands of each tracer medicine registered and therefore legally allowed to be on market in the country.

**Table 2:** **Availability of tracer medicines in private drug outlets surveyed in four districts in Uganda**

**TM**	**All outlets**	***Kampala***	***Mbarara***	***Bundibuygo***	***Apac***	**Outlet type**	
	**(n = 126)**	**(n = 47)**	**(n = 37)**	**(n = 31)**	**(n = 11)**	**Pharmacies**	**Drug shops**
						**(n = 60)**	**(n = 66)**
**ART**	124 (98%)	46 (98%)	37 (100%)	30 (97%)	11 (100%)	59 (98%)	65 (99%)
**MET**	91 (72%)	46 (98%)	26 (70%)	16 (52%)	3 (27%)	56 (93%)	35 (53%)
**OXY**	46 (37%)	21 (45%)	13 (36%)	10 (32%)	2 (18%)	27 (45%)	19 (29%)
**FLU**	42 (33%)	34 (72%)	7 (19%)	1 (3%)	0	42 (70%)	0
**RIF**	12 (10%)	8 (17%)	2 (6%)	2 (7%)	0	11 (18%)	1 (2%)
**LAM**	2 (2%)	2 (4%)	0	0	0	2 (3%)	0

ART = artemisinin-based combinations; MET = metformin; OXY = oxytocin; FLU = fluoxetine; RIF = rifampicin; LAM = lamivudine.

**Table 3:** **Availability of tracer medicine brands listed on the National Drug Register (NDR) as of July 2013 (NDR updated 07/16/2013 and accessed 8/15/2013)**

		**ART**	**MET**	**OXY**	**FLU**	**RIF**	**LAM**
**No. brands on the NDR**		72	40	6	3	9	71
**No. brands in outlets**		13	12	4	4	6	2
**% NDR brands in all outlets**		18%	28%	33%	100%	11%	3%
**Outlet type**	*Pharmacies*	12	12	3*	4*	6*	2
	*Drug shops*	10	6*	2*	0	1	0
**District**	*Kampala*	8	5	2	4*	4*	2
	*Mbarara*	11	10*	2*	3	1	0
	*Apac*	5	4	1	0	0	0
	*Bundibuygo*	7	2	2*	1	1	0
**Brands not on the NDR**		0	1	2	1	5	0

*Location where brands not on NDR were found.

### Ethical considerations

The study protocol was approved by the Institutional Research Committee of Mbarara University of Science and Technology and the Uganda National Council for Science and Technology.

## Results

### Availability of tracer medicines in pharmacies and drug shops

The availability of the six tracer medicines varied by outlet type and location (Table 2). Artemisinin-based combinations and metformin were the most prevalent products. Artemisinin-based combination treatments were found in nearly all pharmacies and drug shops (98%). Metformin was found mainly in urban areas, as was fluoxetine. About 29% of drug shops stocked oxytocin. Rifampicin was also minimally available across medicine outlets and regions; 9.6% of total outlets surveyed. Only 2 pharmacies in Kampala stocked lamivudine, a drug used for the treatment of HIV.

### Registered and unregistered brands

The brands of the six tracer medicines in the surveyed outlets were compared with those registered on the NDR (Table 3). Of the 72 registered brands on the NDR of artemisinin-based combination treatments only 13 were available in outlets (approximately 18%). Of the 40 brands of metformin, 12 were available. All three brands of fluoxetine were available. For oxytocin and rifampicin, 4 out of 6 and 6 out of 9 registered brands were available, respectively. Seventy-one brands of lamivudine are registered with the NDA in Uganda however only 2 were found in pharmacies. Nine unregistered brands were found in 28 medicine outlets (Table 4). Unlisted generic metformin was found in Mbarara drug shops. Pitocin, the oxytocin innovator, was found in 15 pharmacies and drug shops in Mbarara and Bundibugyo. Fluoxetine was only found in a few pharmacies; however, Prozac, the unlisted innovator brand, was found in a pharmacy in Kampala. Five unregistered brands of single drug formulation of rifampicin that originated in Kenya and one from India were found in pharmacies in Kampala. Overall, there were 9 unregistered medicines found in the outlets surveyed; 3 of these medicines were originator brands of oxytocin and fluoxetine. The locations where these unregistered medicines were found are listed in Table 3.

**Table 4:** **List of individual tracer medicine brands available in private drug outlets and frequency of brands available in outlets surveyed**

**TM**	**Formulation & strength**	**Brands**	**License holder**	**Country of manufacture**	**Frequency of brand in total outlets surveyed**	**Frequency of brand in total pharmacies surveyed**	**Frequency of brand in total drug shops surveyed**
					**(n = 126)**	**(n = 60)**	**(n = 66)**
**ART**	*Artemether/Lumefantrine* 20/120 mg (tablet)	Lonart	Bliss GVS Pharma	India	68	38	30
		Coartem (Innovator)	Novartis	USA/Switzerland	56	35	21
		Lumartem	Cipla Ltd/Quality Chemicals	India/Uganda	43	20	23
		Artefan	Ajanta Pharma	India	36	15	21
		Lumether	Astra Life Care	India	9	0	9
		Lumiter	Macleods Pharmaceuticals Ltd	India	2	0	2
	*Artesunate/Mefloquine* 600/750 mg (tablet)	Artequin	Mepha Ltd	Switzerland	2	1	1
	*Artemesinin/Nepthoquin* 250/100 mg (tablet)	Arco	Kunming Pharmaceutical Co	China	11	9	2
	*Artesunate/Amodiaquine* 50/200 mg (tablet)	Artesunate/Amodiaquine Winthrop	Sanofi Aventis	Morocco	2	1	1
		Falcimon-kit	Cipla Ltd	India	2	2	0
	*Dihydroartemisinin/ Piperaquine* 40/320 mg (tablet)	Duo-cotexcin	Beijing Holley Cotec Pharmaceutical Co Ltd	China	17	13	4
		P-alaxin	Bliss GVS Pharma	India	8	6	2
	*Artemether* 100 mg/ml (injection)	Artenam	Arenco Pharmacuetica NV	Belgium	1	0	1
**MET**	*Metformin* 500 mg (tablet)	Glyformin 500	Remedica Ltd	Cyprus	72	49	23
		Glycomet 500	USV Ltd	India	28	22	6
		Metformin-denk 500	Denk Pharma GMPH & CO KG	Germany	17	16	1
		Glucophage (Innovator)	Merck (PTY) Ltd	France & South Africa	13	12	1
		Formin 500*	Stadmed Pvt Ltd	India	4	1	3
		Bigomet 500	Aristo Pharmaceuticals Pvt Ltd	India	2	1	1
		Metchek 500	Indoco Remedies Ltd	India	2	1	1
	*Metformin* 850 mg (tablet)	Glycomet 850	USV Ltd	India	1	0	1
	*Metformin* 1000 mg (tablet)	Glycomet 1000SR	USV Ltd	India	1	1	0
		Metformin denk	Denk Pharma GMPH & CO KG	Germany	1	1	0
		Ranophage OD	Ranbaxy Laboratories Ltd	India	1	1	0
	*Metformin/Glibenclamide*500/5 mg (tablet)	Duotrol	USV Ltd	India	4	4	0
**OXY**	*Oxytocin 5 IU* 5 IU/ml (ampoule)	Oxytocin	Win-Medicare	India	21	21	0
		Pitocin (Innovator)*	Pfizer Ltd	India	1	0	1
	*Oxytocin 10 IU* 10 IU/ml (ampoule)	Pitocin*	Pfizer Ltd	India	14	2	12
		Oxytocin	IDA/Astra Pharma (U) Ltd/Tata (U) Ltd/Gittoes Pharmaceuticals Ltd	China/India/Pakistan	3	3	0
**FLU**	*Fluoxetine hydrochloride* 20 mg (capsule)	Nuzac 20	Cipla Ltd	India	18	18	0
		Fluoxetine	Cadila Healthcare Ltd	India	14	14	0
		Fludac	Cadila Pharmaceuticals Ltd	India	9	9	0
		Prozac (Innovator)*	Eli Lilly & Co	USA	1	1	0
**RIF**	*Rifampicin* 300 mg (tablet)	Unirif 300*	Universal Pharmacy (K) Ltd	Kenya	3	3	0
		Tikocin 300*	Flamingo Pharmaceuticals	Kenya	2	2	0
		Rifacos*	Cosmos Ltd	Kenya	1	1	0
		Rifampicin*	Sino-Kenya Pharmaceuticals Ltd/Panacea Biotech/Cosmos Ltd/El-Nasr Pharm	Kenya	1	1	0
	*Rifampicin/Isoniazid* 150/75 mg (tablet)	Rihide 150	Cosmos Ltd	Kenya	4	4	0
	*Rifampicin/Isoniazid/ Ethambutol/Pyrizinamide* 150/75/275/400 mg (tablet)	ForeCox-Trac 150*	Macleods Pharmaceuticals Ltd	India	1	1	0
**LAM**	*Lamivudine* 150 mg (tablet)	Epivir (Innovator)	Glaxo Wellcome (Kenya) Ltd	South Africa	1	1	0
		Lamivudine	Aurobindo Pharma Ltd/Strides Arcolab Ltd/Mylan Laboratories/Hetero Drugs Ltd/Cipla Ltd	India	1	1	0

*Brands not on the NDR.

## Discussion

Private drug outlets play a major role in selling essential medicines especially when the public health system is unreliable due to issues with procurement or stockouts [23]. The six tracer medicines investigated for this study are first-line treatments for common ailments and should be available free at the point of use in the public sector. The presence of these medicines in the private outlets highlights the existing gaps in the public sector. The presence of Class B drugs in drug shops is indicative of weak regulatory enforcement and lack of access in the public sector.

Pharmacy registration requires that a pharmacy be manned by a licensed pharmacist, a requirement that may not be met especially in rural regions due to the small number of pharmacy professionals in the country, while a drug shop can be registered by any health worker; i.e. nurse, midwife clinical officer [6,24]. Lack of pharmacies, especially in rural areas, together with inadequate enforcement may be responsible for the presence of Class B drugs in drug shops that fill the gap in availability.

### Availability of tracer medicines in pharmacies and drug shops

Uganda had over 12 million suspected cases of malaria in 2011 that led to nearly 500,000 inpatient visits [25]. Almost all outlets surveyed carried artemisinin-based malaria treatments, particularly artemether/lumefantrine, which the Ugandan Clinical Guidelines recommends as the first-line treatment for uncomplicated malaria [26]. Although there is a vertical disease programme for malaria no malaria-specific clinics exists in hospitals, as patients are able to seek malaria treatment at virtually any healthcare facility. The Malaria Control Programme guarantees the availability of antimalarials as part of the national programme for controlling malaria.

Metformin is the most common worldwide treatment of Type 2 Diabetes. The requirement of daily treatment for this chronic condition explains the presence of this medicine in many drug outlets surveyed across all districts.

Oxytocin has many obstetric uses and is listed in the Ugandan Clinical Guidelines for induction of labour in certain circumstances or management of post-partum haemorrhage. It requires cold storage and only available as an injectable and therefore requires specific storage conditions and trained personnel to deliver the medication. The presence of oxytocin at drug shops is of major concern, as they have no facility for cold storage.

Fluoxetine has recently been added to Uganda’s EML in 2012, but has not yet been added to the treatment guidelines. This survey found fluoxetine only available in urban pharmacy outlets suggesting treatments for depression are only just beginning to enter the market place in Uganda.

Treatments for TB, HIV, and malaria are managed by the Ministry of Health through national drug programmes with separate clinics in public hospitals handling HIV and TB patients. In some cases joint HIV and TB clinics exist to simplify treatment regimens for patients.

Rifampicin is a powerful antibiotic and should not be purchased as a single formulation. Ugandan policy states rifampicin should only be used to treat TB and leprosy and ideally as part of a combination therapy. The use of rifampicin is restricted to combination treatment regimens to prevent antibacterial resistance from developing. Two-, 3- or 4-medicine fixed dose combinations that include rifampicin are available for the treatment of tuberculosis and are listed as such in the EMLU. Very few outlets surveyed carried rifampicin products. This could indicate strong support for programme medicines being available freely in the public healthcare system or widespread stockouts at the time the survey was conducted. It could be found in drug shops in order to treat other infections such as brucellosis.

Treatment of HIV infection and prevention of mother to child transmission both utilise lamivudine as part of a combination therapy and therefore it is listed on the EMLU as both a single formulation and a component of fixed-dose combinations. Because the majority of antiretrovirals now are combined formulations, primarily as a means of limiting pill burden, lamivudine monotherapy stocks are limited. Therefore lamivudine was not found in many outlets possibly due to its availability in the public sector through vertical programmes, restrictions on pharmacies preventing them from stocking antiretrovirals, or medicine stockouts during the survey. Lamivudine monotherapy can also used to treat Hepatitis B infection, which may also explain some of the availability.

### Availability of different brands of tracer medicines in pharmacies and drug shops

The NDA has registered many brands, most of which were produced in India and the USA. The absence of African-produced brands, with the exception of South African metformin and lamivudine and Kenyan rifampicin (Table 4), may be due to consumer preferences for overseas products due to quality perceptions (*fieldwork notes; Sep 2011 – Oct 2012*). Only a small percentage of brands of the tracer medicines listed on the drug registry were actually found in our outlet survey (Table 3). The highest number of registered brands was for artemisinin-based combinations and lamivudine, both of which are available through vertical programmes that are freely available to the public.

### Availability of Non registered brands in pharmacies and drug shops

Registration of medicines with the NDA is a legal requirement however unregistered products were found in surveyed drug shops and pharmacies including: 1 fluoxetine, 1 metformin, 2 oxytocin, and 5 rifampicin. In this study it is mainly brands of rifampicin, a highly regulated drug, that were unregistered. Unregistered brands of rifampicin were found in 6 pharmacies surveyed in Kampala (13% of total) and 1 pharmacy each in Mbarara and Bundibugyo. Pitocin was found in 20% of drug shops surveyed in Bundibugyo and 25% of drug shops surveyed in Mbarara. None of which had the facilities for cold chain storage. The presence of unauthorised drugs on the market has been attributed to the illegal operation of drug outlets [12]. Uganda‘s porous borders allow illegal products to reach the market.

## Limitations

Medicine registration data were limited to the NDA’s online database and the time when it was accessed in July 2013. An accurate number of drug shops in each district were not readily available so the sampling framework was based on an approximation, which may result in a biased sample and therefore not be truly representative. The availability of medicines refers only to the day of data collection; no assumptions of availability over time can be determined from this study.

## Conclusions

Few studies have investigated the availability of essential medicines in private medicine outlets. This study shows that some essential medicines are quite common in private outlets and sold in drug shops that do not have a license for sale. In addition, only a few of the brands on the NDR were present in outlets surveyed. The presence of unauthorised formulations indicates weak enforcement. Most of the available products were imported from India; and locally manufactured products were not common.

In order to increase access to safe and effective essential medicines throughout the private sector in Uganda, three areas can be improved upon. Firstly, enhance and strengthen the regulation and enforcement of valid licensing of pharmacies and drug shops specifically in respect to the class of drugs being offered. Secondly, review medicine registration procedures within the NDA and ensure the NDR is kept up-to-date and review the need for so many brands. Lastly, investigate the reasons why patients are using the private sector to obtain certain essential medicines that should be available free in public facilities and what should be done about this.

## Abbreviations

EML: Essential medicines list
EMLU: Essential medicines list of Uganda
NDA: National Drug Authority
NDR: National drug register
TM: Tracer medicine
